# Endotoxin Pretreatment Mitigates Myocardial Ischemia-Reperfusion Injury Through Preservation of Mitochondrial Respiration: A Combined Assessment of In Vivo, Ex Vivo, and In Vitro Data

**DOI:** 10.3390/ijms262211162

**Published:** 2025-11-19

**Authors:** Reverien Habimana, Jiae Seong, YeongEun Jo, Ryul-Hee Kim, Hyo-Jung Kim, Kyung Soon Choi, Mukhammad Kayumov, Francis O. Obiweluozor, Wang-In Kim, Hwa Jin Cho, Dowan Kim, Kook Joo Na, Inseok Jeong

**Affiliations:** 1 Department of Medical Science, Chonnam National University Graduate School, Gwangju 61469, Republic of Korea; habire04@gmail.com; 2 Extracorporeal Cardiopulmonary Innovation, Technology, and Education (EXCITE) Research Group, Chonnam National University Hospital, Gwangju 61469, Republic of Korea; 24p01@naver.com (J.S.); yeongeun7738@gmail.com (Y.J.); rlafbfgml125@gmail.com (R.-H.K.); rlagywjd198@naver.com (H.-J.K.); queen050178@naver.com (K.S.C.); mkayumov@bwh.harvard.edu (M.K.); francismoore042@gmail.com (F.O.O.); wangto9@naver.com (W.-I.K.); chhj98@gmail.com (H.J.C.); maskjoa@hanmail.net (D.K.); 3 Division of Transplant Surgery, Department of Surgery, Brigham and Women’s Hospital, Harvard Medical School, Boston, MA 02115, USA; 4 Department of Pediatrics, Chonnam National University Children’s Hospital and Medical School, Gwangju 61469, Republic of Korea; 5 Department of Thoracic and Cardiovascular Surgery, Chonnam National University Hospital and Medical School, Gwangju 61469, Republic of Korea

**Keywords:** acute myocardial infarction, endotoxin pretreatment, ischemia–reperfusion injury, mitochondrial respiration

## Abstract

Acute myocardial infarction is the most common form of coronary artery disease, and myocardial ischemia–reperfusion injury remains a major challenge despite advances in reperfusion therapy. Endotoxin preconditioning has been linked to reduced ischemia–reperfusion injury, but mechanisms remain unclear, and prior studies have used varied assessment methods with inconsistent results. In this study, we confirmed the protective effect of endotoxin preconditioning and assessed its role in preserving mitochondrial respiration using a multi-model approach of in vivo ischemia–reperfusion rat model, ex vivo normothermic rat heart perfusion, and in vitro hypoxia–reoxygenation in neonatal rat cardiomyocytes. Hemodynamic and cell-based analyses were performed in control (*n* = 5), ischemia–reperfusion/hypoxia–reoxygenation (*n* = 4/3), and endotoxin-pretreated (*n* = 5/3) groups. Low-dose endotoxin pretreatment significantly preserved left ventricular function, myocardial oxygen consumption, and mitochondrial respiration (*p* < 0.001). Preservation of function was associated with reduced hypoxia-inducible factor 1-alpha (HIF-1α) expression and decreased mitochondrial superoxide production, indicating reduced oxidative stress. Nonlethal endotoxin pretreatment protects the myocardium from ischemia–reperfusion injury by sustaining mitochondrial respiration and limiting oxidative damage. These findings support further investigation in large animal models to better replicate human myocardial infarction and evaluate translational potential.

## 1. Introduction

Acute myocardial infarction (AMI), the most common manifestation of coronary artery disease (CAD), is a major cause of morbidity and mortality worldwide, accounting for over 10% of annual deaths [1,2,3]. Its incidence exceeds half a million new cases annually in developed countries and is rising in developing nations [4,5]. Although AMI has a multifactorial etiology, rupture of atherosclerotic plaques resulting in an abrupt reduction in coronary blood flow remains the primary cause [6,7]. Over the past three decades, AMI management has advanced considerably, with the main therapeutic goal being rapid reperfusion of ischemic myocardium before irreversible infarction. This is achieved through urgent interventions such as percutaneous coronary intervention (PCI) or intravenous thrombolytic therapy [8]. Despite these advances, current strategies cannot fully restore or maintain optimal hemodynamics in all patients. Furthermore, reperfusion itself often triggers myocardial ischemia–reperfusion injury (IRI), which can worsen tissue damage [9,10]. The inability of existing treatments to completely recover myocardial function highlights the urgent need for novel translational approaches aimed at preventing the transition from myocardial infarction to adverse ventricular remodeling and heart failure [11,12]. Sepsis preconditioning has been demonstrated to confer cardioprotection against ischemia–reperfusion (I/R) injury in both ex vivo isolated perfused heart models [13], and in vivo experimental settings [14]. Similarly, administration of a sublethal dose of endotoxin has been shown to mitigate hypoxia–reoxygenation (H/R) injury in cardiomyocytes [15]. Despite these observations, the precise physiological mechanism underlying endotoxin-induced myocardial protection remains incompletely understood. Current evidence suggests that preservation of metabolic function during myocardial ischemia involves multiple signaling pathways [16,17], with a pivotal role attributed to the activation of adenosine monophosphate-activated protein kinase (AMPK). AMPK activation attenuates I/R-induced myocardial injury primarily by suppressing excessive reactive oxygen species (ROS) production and restoring cellular energy homeostasis [18,19]. To sustain continuous cardiac contractile function, cardiomyocytes require a constant supply of cellular energy, with over 90% of this energy generated by mitochondria through oxidative phosphorylation in the form of adenosine triphosphate (ATP) [20,21,22,23]. Disruption of mitochondrial energy metabolism by oxidative stress is widely recognized as a central mechanism underlying ischemia–reperfusion (I/R)-induced myocardial injury [24,25,26]. Consequently, preserving or restoring mitochondrial function represents a promising strategy to enhance myocardial recovery following cardiac events such as I/R injury, potentially offering novel translational targets for the management of myocardial infarction (MI). However, its role in the context of endotoxin preconditioning has not yet been thoroughly investigated.

This study aims to validate the previously reported cardioprotective effects of endotoxin preconditioning against myocardial I/R injury and to elucidate its impact on mitochondrial respiration and cellular energy metabolism. Specifically, we sought to determine whether endotoxin preconditioning preserves mitochondrial oxidative phosphorylation capacity, maintains ATP production, and reduces oxidative stress–induced impairment of mitochondrial function during ischemia and reperfusion. By integrating in vivo, ex vivo, and in vitro experimental models, we further aimed to characterize the extent to which these mitochondrial effects contribute to the observed functional preservation of the myocardium following I/R injury.

## 2. Results

### 2.1. Exclusions

No mortality occurred following LPS administration. Of the fifteen rats used in the in vivo experiments, ten underwent temporary left anterior descending artery (LAD) ligation (five in the I/R group and five in the I/R + LPS group). Two rats (one from each of these groups) were excluded from data analysis due to ventricular fibrillation or severe bleeding during LAD occlusion or reperfusion. In the ex vivo experiments, two hearts from the I/R group were excluded because of irreversible arrhythmias before ischemia or a coronary flow rate < 8 mL/min during the equilibration period. The final sample sizes included in the analyses were as follows: in vivo Control (*n* = 5), I/R (*n* = 4), and I/R + LPS (*n* = 4); ex vivo Control (*n* = 5), I/R (*n* = 3), and I/R + LPS (*n* = 5); in vitro Control (*n* = 3), H/R (*n* = 3), and H/R + LPS (*n* = 3).

### 2.2. Endotoxin Pretreatment Preserved Left Ventricular Function and Myocardial Oxygen Consumption Following Ischemia-Reperfusion In Vivo

**Figure 1:**
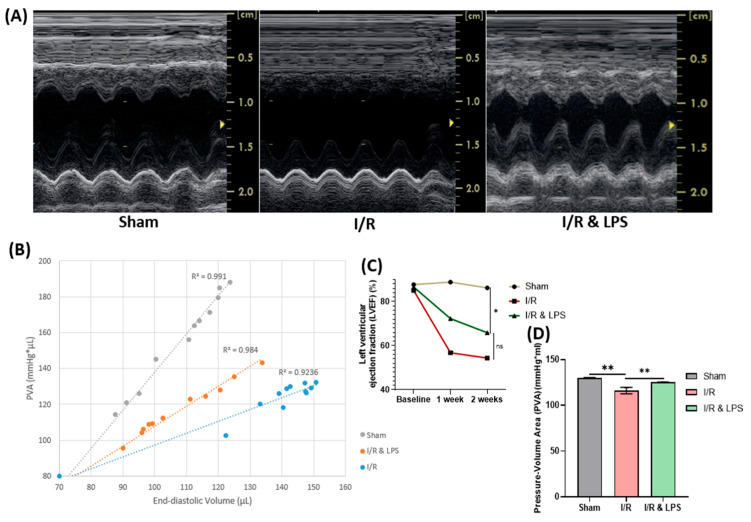
Effect of endotoxin preconditioning on left ventricular ejection fraction and myocardial oxygen consumption after ischemia and reperfusion. (**A**) Representative echocardiographic images. (**B**) Pressure–Volume Area (PVA) scatter plots with linear trendlines. (**C**) Quantitative analysis of Left Ventricular Ejection Fraction (LVEF, %) and (**D**) PVA (mmHg·µL) in Sham, ischemia/reperfusion (I/R; 30 min ischemia + 2 weeks reperfusion), and I/R with endotoxin preconditioning (I/R + LPS) groups (*n* = 5 per group). I/R = ischemia/reperfusion; I/R + LPS = ischemia/reperfusion with endotoxin preconditioning; LPS = lipopolysaccharide. Data are presented as mean ± standard deviation. Statistical analysis was performed using one-way ANOVA with Tukey’s post hoc test. ns = not significant (*p* ≥ 0.05); * *p* < 0.05; ** *p* < 0.01.

Cardiac functional assessment performed to determine whether endotoxin preconditioning confers protection against ischemia–reperfusion–induced systolic dysfunction showed that Left Ventricular Ejection Fraction (LVEF) and Fractional Shortening (FS) were significantly higher in the endotoxin-pretreated group compared to the ischemia–reperfusion (I/R)-only group, indicating preserved left ventricular systolic function (*p* < 0.01; Figure 1A). Hemodynamic assessment using pressure-volume (PV) loop analysis demonstrated that endotoxin pretreatment improved several key parameters of cardiac function. Notably, the pressure-volume area (PVA), an index representing the total mechanical energy generated by ventricular contraction and closely related to myocardial oxygen consumption, was significantly increased (*p* < 0.01; Figure 1B). Additionally, stroke work (SW), a measure of the work performed by the heart during each beat, was improved in the endotoxin-pretreated group. End-diastolic volume (EDV) and end-systolic volume (ESV), which reflect ventricular filling and contractility, respectively, were favorably modulated, suggesting improved ventricular compliance and systolic performance. Collectively, these data confirm that endotoxin pretreatment significantly preserves left ventricular contractile function and optimizes myocardial energetics after I/R injury.

### 2.3. Endotoxin Pretreatment Reduced Myocardial Fibrosis and Inflammation Following Ischemia–Reperfusion In Vivo

**Figure 2:**
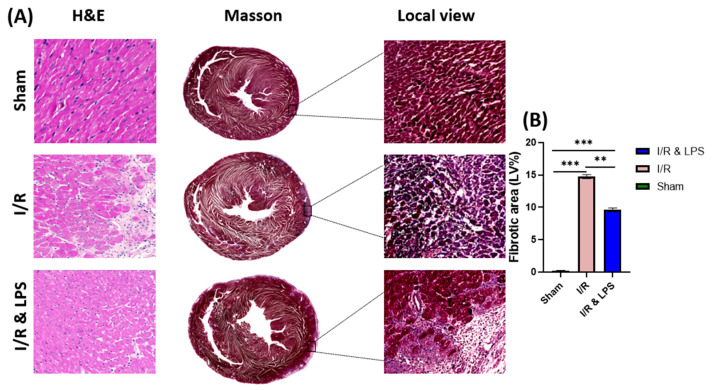
Effect of Endotoxin Preconditioning on Myocardial Fibrosis Following Ischemia–Reperfusion. (**A**) Representative histological images of hematoxylin and eosin (H&E) and Masson’s trichrome-stained left ventricular sections from Sham, ischemia/reperfusion (I/R), and endotoxin preconditioning + ischemia/reperfusion (I/R & LPS) groups (*n* = 5 per group). Scale bar = 200 μm. (**B**) Quantification of fibrotic area expressed as a percentage (%) of total myocardial tissue area. Data are presented as mean ± standard deviation (SD). Statistical analysis was performed using one-way ANOVA followed by Tukey’s post hoc test for multiple group comparisons. ** *p* < 0.01, *** *p* < 0.001 versus I/R group. Abbreviations: I/R, ischemia/reperfusion; LPS, lipopolysaccharide.

To further evaluate the cardioprotective effects of endotoxin preconditioning, histological analyses were performed to assess myocardial remodeling and inflammation following ischemia–reperfusion injury. Pretreatment with endotoxin before ischemia–reperfusion (I/R) injury significantly preserved the structural integrity and functional capacity of the left ventricle by attenuating both myocardial fibrosis and inflammatory responses (Figure 2). Histological examination using Masson’s trichrome staining demonstrated a substantial reduction in collagen deposition, a hallmark of fibrotic remodeling, in endotoxin-pretreated hearts compared to non-treated I/R controls (Figure 2A). This decrease in fibrotic tissue accumulation was accompanied by preservation of overall myocardial architecture, with notably fewer disruptions and areas of necrosis in the endotoxin group. Additionally, hematoxylin and eosin (H&E) staining revealed a marked reduction in neutrophil infiltration and inflammatory cell density within the infarct border zones of pretreated hearts, indicating an attenuated inflammatory response. These findings suggest that endotoxin pretreatment effectively mitigates the maladaptive fibrotic remodeling and excessive inflammatory cell recruitment typically induced by I/R injury, thereby contributing to improved myocardial healing and functional recovery in the post-ischemic period.

### 2.4. Endotoxin Pretreatment Preserved Ventricular Function Following Ischemia and Reperfusion in Ex Vivo Normothermic Perfusion

**Figure 3:**
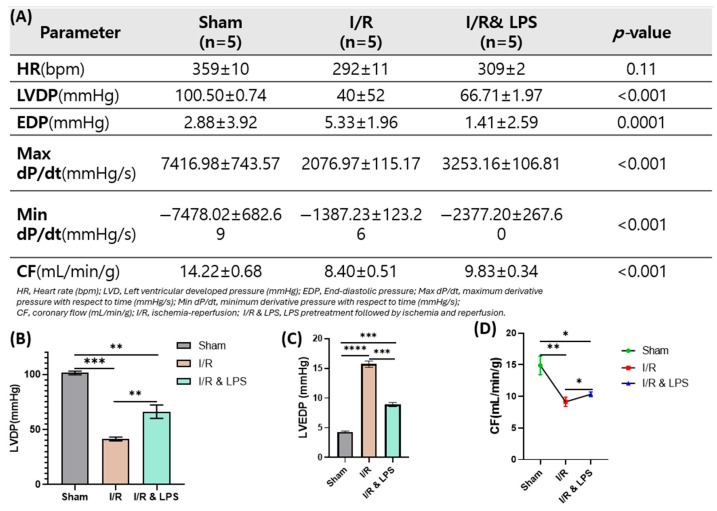
Effect of endotoxin pretreatment on left ventricular function following ischemia–reperfusion under isolated normothermic perfusion conditions. (**A**) Representative table summarizing measured cardiac parameters. Quantitative analysis of (**B**) left ventricular developed pressure (LVDP, mmHg), (**C**) heart rate (HR, bpm), and (**D**) coronary flow (CF, mL/min/g) in Sham, ischemia/reperfusion (I/R; 30 min ischemia + 45 min reperfusion), and endotoxin preconditioning + ischemia/reperfusion (I/R & LPS) groups (*n* = 5 per group). Abbreviations: I/R, ischemia/reperfusion; LPS, lipopolysaccharide. Data are presented as mean ± standard deviation (SD). Statistical comparisons were performed using one-way ANOVA followed by Tukey’s post hoc test. ns, not significant (*p* ≥ 0.05); * *p* < 0.05; ** *p* < 0.01; *** *p* < 0.001; **** *p* < 0.0001 versus I/R group.

To further validate the cardioprotective effects of endotoxin preconditioning under controlled conditions, an ex vivo normothermic perfused rat heart model was employed to assess post-ischemic cardiac performance. In this model, endotoxin preconditioning significantly preserved key indicators of cardiac function after ischemia–reperfusion injury. Compared to ischemia/reperfusion-only hearts, endotoxin-pretreated hearts exhibited notably higher Left Ventricular Developed Pressure (LVDP), indicating improved contractile strength during reperfusion (*p* < 0.001; Figure 3B). Coronary flow (CF), an indicator of myocardial perfusion and coronary vascular integrity, was also significantly enhanced in the endotoxin group, suggesting preserved microvascular function and vasodilation capacity. Additionally, endotoxin pretreatment markedly improved both the maximum and minimum derivatives of left ventricular pressure over time (Max dP/dt and Min dP/dt, respectively), reflecting superior systolic contractility and diastolic relaxation (*p* < 0.001 for both parameters). These functional improvements demonstrate that endotoxin preconditioning enhances myocardial functional recovery and coronary perfusion following ischemia–reperfusion injury in the isolated heart model by preserving intrinsic myocardial performance and coronary circulation under controlled ex vivo conditions, independent of systemic neurohumoral influences.

### 2.5. Identification of Isolated Primary Cardiomyocytes

**Figure 4:**
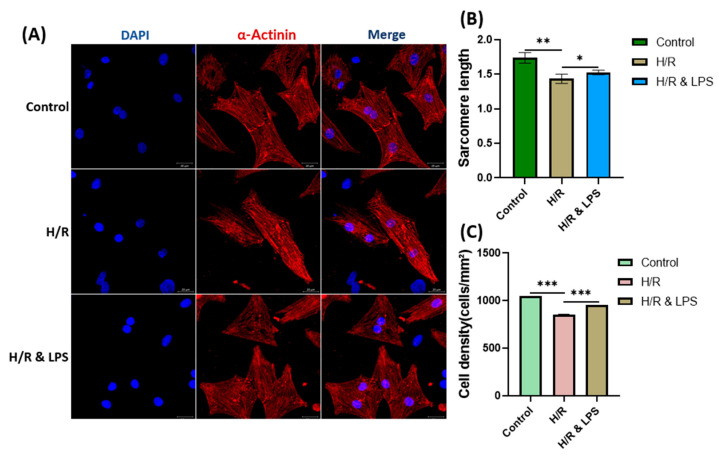
Effect of endotoxin preconditioning on cardiomyocyte structure after hypoxia/reoxygenation injury. (**A**) Representative confocal microscopy images of neonatal primary rat cardiomyocytes in the control, 4 h hypoxia + 2 h reoxygenation (H/R), and endotoxin preconditioning + H/R (H/R & LPS) groups (*n* = 3 per group). Cells were immunostained for sarcomeric α-actinin (red) and counterstained with DAPI for nuclei (blue). Scale bar: 20 μm. (**B**) Sarcomere length and (**C**) cell density quantification. H/R = hypoxia/reoxygenation; H/R & LPS = hypoxia/reoxygenation with endotoxin preconditioning; LPS = lipopolysaccharide. Data are presented as mean ± standard deviation. One-way ANOVA followed by Tukey’s post hoc test was used for multiple group comparisons. * *p* < 0.05; ** *p* < 0.01; *** *p* < 0.001.

To investigate the cellular mechanisms underlying the cardioprotective effects of endotoxin preconditioning, primary neonatal rat cardiomyocytes were isolated and subjected to hypoxia–reoxygenation (H/R) injury. Immunocytochemical staining revealed that more than 95% of the isolated primary cells were positive for sarcomeric α-actinin, confirming their identity as cardiomyocytes. This verification was performed for all experimental groups before further analyses. Morphological assessment demonstrated that endotoxin-preconditioned cardiomyocytes maintained structural integrity after hypoxia–reoxygenation (H/R) injury. Sarcomere length measurements served as a quantitative indicator of contractile apparatus preservation, with values in the LPS-pretreated group approaching those of normoxic controls. This finding supports the protective effect of endotoxin preconditioning in mitigating H/R-induced contractile damage. Cell density analysis indicated reduced viability or proliferation under hypoxic conditions. While LPS-pretreated cells exhibited slightly reduced density compared to normoxic controls, their density remained markedly higher than in the untreated H/R group, suggesting partial preservation of cell viability (Figure 4C). In normoxic controls, cardiomyocytes displayed well-aligned, periodic sarcomeric striations, consistent with intact and functional contractile structures. Correspondingly, Fast Fourier Transform (FFT) analysis of sarcomere line intensity profiles revealed distinct, sharp peaks in the frequency domain, indicative of regular sarcomeric spacing. In contrast, untreated hypoxic cardiomyocytes exhibited disrupted sarcomeric organization, with FFT spectra showing diffuse or blurred peaks and central frequency smearing, consistent with structural disarray and impaired contractility. Strikingly, endotoxin-preconditioned cells displayed restored FFT spectral sharpness with well-defined frequency components, indicating reestablishment of sarcomeric order and functional preservation of contractile elements following H/R injury.

### 2.6. Endotoxin Pretreatment Preserved Cardiomyocyte Viability After Hypoxia–Reoxygenation

**Figure 5:**
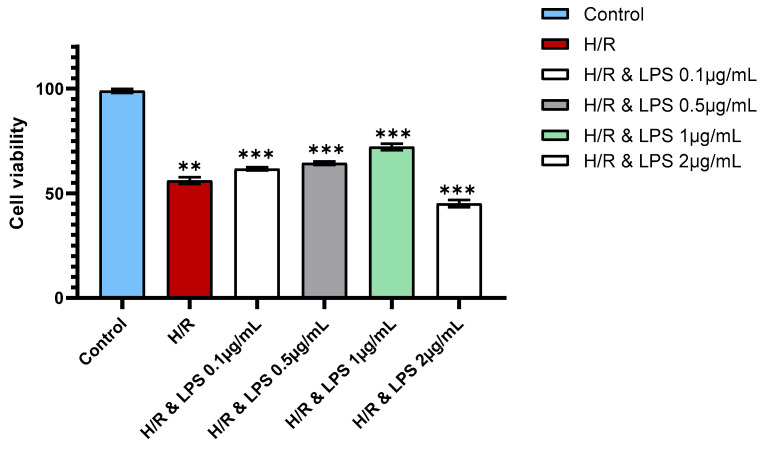
Effect of different endotoxin dosages on cardiomyocyte viability following hypoxia–reoxygenation. Representative bar graph showing cell viability of primary neonatal rat cardiomyocytes in control, 4 h hypoxia + 2 h reoxygenation (H/R), and endotoxin-preconditioned H/R groups treated with varying concentrations of lipopolysaccharide (LPS: 0.1 (*p* = 0.0008), 0.5, 1.0, and 2.0 µg/mL) (*n* = 3 per group). H/R = hypoxia–reoxygenation, H/R & LPS = hypoxia–reoxygenation with endotoxin preconditioning. Data are presented as mean ± standard deviation. Statistical analysis was performed using one-way ANOVA followed by Tukey’s post hoc test for multiple comparisons. ** *p* < 0.01; *** *p* < 0.001 versus untreated H/R group.

Having confirmed the structural preservation of cardiomyocytes following endotoxin preconditioning, we next examined whether this intervention also enhanced cell survival under hypoxic stress. LPS preconditioning significantly improved the survival of cardiomyocytes subjected to hypoxia–reoxygenation compared with untreated cells. Among the tested concentrations, pretreatment with 1 µg/mL LPS resulted in the highest viability, which was significantly greater than that observed with 0.1 µg/mL or 0.5 µg/mL LPS (*p* < 0.001; Figure 5). In contrast, exposure to a higher concentration of 2 µg/mL LPS led to a marked reduction in viability compared with the 1 µg/mL group (*p* < 0.001). These findings suggest a dose-dependent effect in which low-dose endotoxin pretreatment (particularly 1 µg/mL) confers optimal cytoprotection, whereas excessive doses impair cell survival. This biphasic response highlights the importance of dose optimization when applying endotoxin preconditioning as a cardioprotective strategy.

### 2.7. Endotoxin Pretreatment Reduced Hypoxia in Neonatal Rat Cardiomyocytes

**Figure 6:**
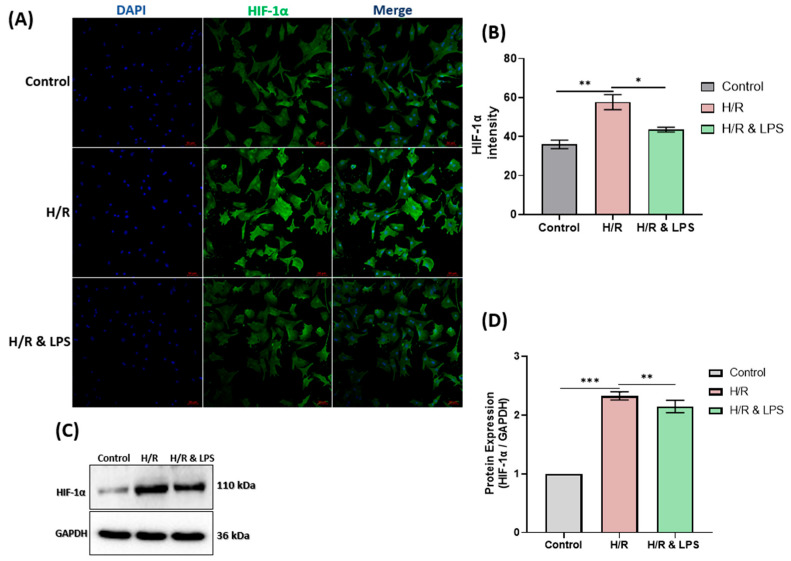
Effect of endotoxin preconditioning on hypoxia levels in neonatal rat cardiomyocytes after hypoxia–reoxygenation. (**A**) Representative confocal microscopy images showing HIF-1α (green) and nuclei (DAPI, blue) staining. (**B**) Quantification of HIF-1α fluorescence intensity. (**C**) Representative Western blot images of HIF-1α expression. (**D**) Densitometric analysis of Western blot data. Experimental groups: normoxic control, 4 h hypoxia + 2 h reoxygenation (H/R), and endotoxin preconditioning + H/R (H/R & LPS) (*n* = 3 per group). Scale bar = 20 μm. H/R = hypoxia/reoxygenation, H/R & LPS = hypoxia/reoxygenation with endotoxin preconditioning, LPS = lipopolysaccharide. Data are presented as mean ± SD. One-way ANOVA followed by Tukey’s post hoc test was used for statistical comparisons. * *p* < 0.05, ** *p* < 0.01, *** *p* < 0.001.

Building on the observed improvements in cardiomyocyte survival, we next assessed whether endotoxin preconditioning alleviates intracellular hypoxia during hypoxia–reoxygenation. Endotoxin-preconditioned cardiomyocytes (H/R & LPS) exhibited markedly reduced hypoxia compared with untreated hypoxic cells, as evidenced by both immunohistochemistry and Western blot analysis of Hypoxia-Inducible Factor 1-alpha (HIF-1α) (Figure 6A,C). Immunohistochemistry showed a visibly lower HIF-1α staining intensity (*p* < 0.01), while Western blot quantification revealed a significant reduction in HIF-1α protein expression (*p* < 0.001) compared with the hypoxia–reoxygenation (H/R) group. Hypoxia–reoxygenation significantly increased HIF-1α expression relative to normoxic controls (*p* < 0.001), whereas LPS pretreatment effectively attenuated this upregulation (*p* < 0.05; *n* = 3). These findings indicate that endotoxin preconditioning confers resistance to hypoxia–reoxygenation-induced injury by mitigating cellular hypoxia.

### 2.8. Endotoxin Pretreatment Reduces Mitochondrial Oxidative Stress and Preserves Cardiac Mitochondrial Respiration Following Hypoxia–Reoxygenation

**Figure 7:**
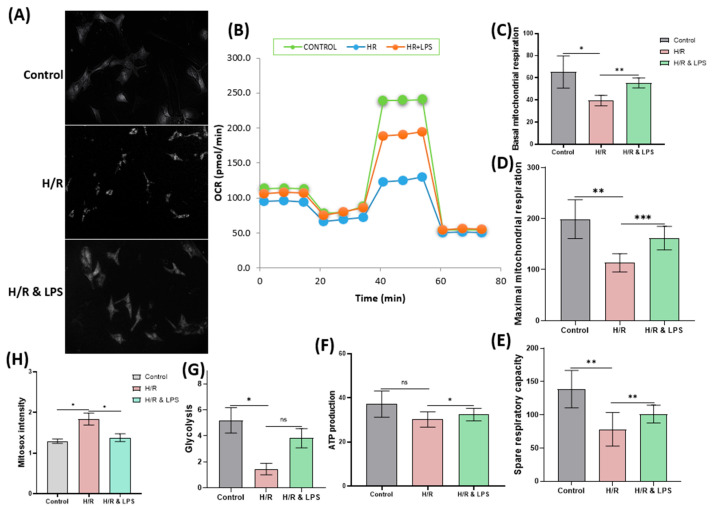
Effect of endotoxin preconditioning on oxidative stress and mitochondrial respiration following ischemia and reoxygenation in neonatal rat cardiomyocytes. (**A**) Representative fluorescence microscopy images stained with MitoSOX™ Green. (**B**) Mitochondrial oxygen consumption rate (OCR) plots. Statistical data showing (**C**) basal mitochondrial respiration, (**D**) maximal mitochondrial respiration, (**E**) spare respiratory capacity, (**F**) adenosine triphosphate (ATP) production, (**G**) glycolysis, and (**H**) mitochondrial superoxide (MitoSOX™ Green) intensity. Groups include control, 4 h of hypoxia + 2 h of reoxygenation (H/R), and endotoxin preconditioning + 4 h of hypoxia + 2 h of reoxygenation (H/R & LPS) (*n* = 3 per group). Scale bar: 50 μm. Data are presented as mean ± standard deviation. One-way ANOVA followed by Tukey’s post hoc test was used for statistical comparisons. ns = not significant (*p* ≥ 0.05); * *p* < 0.05; ** *p* < 0.01; *** *p* < 0.001. Abbreviations: H/R = hypoxia/reoxygenation; LPS = lipopolysaccharide.

After confirming that endotoxin preconditioning alleviates cellular hypoxia, we next examined its effects on mitochondrial bioenergetics to evaluate how metabolic function was preserved under hypoxia–reoxygenation stress. Cardiomyocytes pretreated with endotoxin demonstrated significant preservation of basal respiration, maximal respiration, spare respiratory capacity, and ATP production compared to untreated cells subjected to hypoxia-reoxygenation (*p* < 0.01 for mitochondrial respiration parameters; *p* < 0.05 for ATP production; Figure 7C–F). Additionally, glycolytic activity was significantly maintained in the endotoxin-preconditioned group during hypoxia and reoxygenation (*p* < 0.05). Overall, endotoxin pretreatment resulted in the highest levels of basal and maximal mitochondrial respiration, spare respiratory capacity, ATP generation, and glycolytic function, highlighting improved cellular bioenergetics under stress conditions. Consistent with these findings, hypoxia–reoxygenation markedly increased mitochondrial superoxide production, as measured by MitoSOX™ Green fluorescence, in untreated cardiomyocytes. In contrast, the endotoxin-pretreated group exhibited substantially lower mitochondrial superoxide levels (*p* < 0.05; Figure 7H), indicating a significant reduction in mitochondrial oxidative stress induced by endotoxin pretreatment. These results suggest that endotoxin preconditioning effectively mitigates oxidative damage and preserves mitochondrial function in cardiomyocytes exposed to hypoxia–reoxygenation injury.

### 2.9. Endotoxin Pretreatment Reduced Apoptosis Following Hypoxia and Reoxygenation

**Figure 8:**
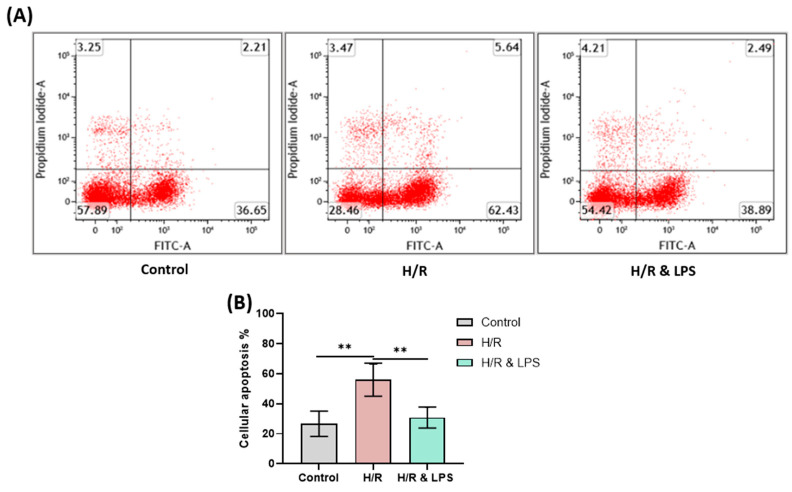
Effect of endotoxin preconditioning on cellular apoptosis after ischemia and reoxygenation. (**A**) Representative flow cytometry images and (**B**) statistical data depicting the percentage of apoptotic cells in primary neonatal rat cardiomyocytes from control, 4 h of hypoxia + 2 h of reoxygenation (H/R), and endotoxin preconditioning + 4 h of hypoxia + 2 h of reoxygenation (H/R & LPS) groups (*n* = 3 per group). H/R = hypoxia/reoxygenation, H/R & LPS = hypoxia/reoxygenation with endotoxin preconditioning, LPS = Lipopolysaccharide. Data are presented as mean ± standard deviation. Statistical comparisons among multiple groups were performed using one-way ANOVA followed by Tukey’s post hoc test. ** *p* < 0.01.

Having established that endotoxin preconditioning preserves mitochondrial function and energy metabolism, we next investigated whether these effects translated into reduced apoptotic cell death following hypoxia–reoxygenation injury. Flow cytometric analysis revealed a significant reduction in both early and late apoptotic cardiomyocytes following endotoxin preconditioning before hypoxia–reoxygenation injury. Specifically, the proportion of apoptotic cells in the endotoxin-pretreated group decreased by approximately 26.26% compared to the untreated hypoxia–reoxygenation group (Figure 8A, *p* < 0.01). This marked reduction highlights a cytoprotective effect of endotoxin preconditioning on cardiomyocytes exposed to hypoxic stress and subsequent reoxygenation. The observed anti-apoptotic effect of endotoxin pretreatment may be attributed to the preserved mitochondrial respiration and decreased mitochondrial superoxide production in preconditioned cardiomyocytes. Since mitochondria play a pivotal role in regulating intrinsic apoptotic pathways, maintenance of mitochondrial integrity likely contributed to the reduction in apoptosis. By mitigating mitochondrial dysfunction and reactive oxygen species (ROS) generation, endotoxin preconditioning prevented the release of cytochrome c and downstream activation of caspases, which are central mediators of apoptosis. Overall, these findings support the potential of endotoxin preconditioning to attenuate hypoxia–reoxygenation-induced apoptotic cell death through a multifaceted mechanism involving the modulation of inflammatory signaling, preservation of mitochondrial function, and oxidative stress reduction. This cytoprotective effect contributes to enhanced cardiomyocyte survival and improved cardiac recovery following ischemia–reperfusion injury.

## 3. Discussion

The present study provides strong evidence that low-dose endotoxin preconditioning confers robust cardioprotection against ischemia–reperfusion (I/R) injury by preserving left ventricular function, limiting structural damage, and promoting cellular survival. Using an integrated approach across in vivo, ex vivo, and in vitro models, we demonstrate that endotoxin pretreatment enhances myocardial contractility and reduces apoptosis, thereby mitigating both the functional impairment and molecular injury associated with I/R. Moreover, our findings confirm that this protective effect is mediated through attenuation of oxidative stress and preservation of mitochondrial respiration. This preservation of mitochondrial function supports sustained oxygen consumption, which is essential for cellular energy metabolism and ultimately ensures structural integrity and survival. Our findings are consistent with previous reports highlighting the protective role of sepsis preconditioning in myocardial ischemia–reperfusion (I/R) injury [27,28,29]. For example, Walshe et al. demonstrated that endotoxin pre-exposure not only preserved cardiomyocyte function under hypoxia/reperfusion-induced stress but also conferred protection to other cell types, suggesting that the benefits of sepsis preconditioning extend beyond the myocardium [13]. Similarly, Merry et al. reported that endotoxin pretreatment significantly attenuated I/R injury in lung tissue [30]. Collectively, these studies, together with our results, underscore the broad cytoprotective potential of endotoxin preconditioning across diverse ischemic settings. The cardioprotective mechanisms of endotoxin preconditioning are multifaceted, encompassing modulation of inflammatory signaling, attenuation of oxidative stress, and preservation of mitochondrial function. Recent research has uncovered key connections between adaptive immunity and atherosclerosis, particularly through immune recognition of low-density lipoproteins (LDL) [31,32,33]. In parallel, toll-like receptors (TLRs) have been identified as crucial mediators in myocardial ischemia–reperfusion injury and subsequent cardiac repair [34,35], underscoring the need for deeper investigation of adaptive and innate immune pathways in both ischemic and septic heart injury. At low doses, endotoxin induces mild activation of toll-like receptor 4 (TLR4) and suppresses excessive NLRP3 inflammasome signaling in cardiomyocytes and immune cells, thereby initiating a controlled inflammatory response that promotes tolerance to subsequent ischemic stress [36,37]. This adaptive response further enhances the expression of cytoprotective proteins, including heat shock proteins (HSPs) and inducible nitric oxide synthase (iNOS), which are both pivotal in limiting oxidative damage, maintaining mitochondrial integrity, and reducing ischemia–reperfusion injury [38,39]. The preservation of mitochondrial respiration in murine hearts following endotoxin preconditioning represents a key novel finding of this study. Our findings of preserved basal and maximal mitochondrial respiration, along with reduced mitochondrial superoxide levels, are consistent with Chen et al., who demonstrated that heart preconditioning with polycytidylic acid (Poly I:C), a synthetic analog of double-stranded RNA that strongly activates antiviral immune responses, protected against myocardial ischemia/reperfusion injury through modulation of inflammatory responses and apoptosis [40]. Furthermore, the protective effect induced by endotoxin preconditioning in this study may be comparable to that of ischemic preconditioning, which is known to inhibit the opening of the mitochondrial permeability transition pore (mPTP), a critical trigger of mitochondrial swelling, loss of membrane potential, and apoptosis [41,42]. By preventing mPTP opening, mitochondrial integrity and function are maintained, thereby reducing apoptotic cell death [43,44], consistent with the decreased apoptosis observed in our study. Collectively, these preconditioning mechanisms converge to limit myocardial injury, preserve contractile function, and enhance recovery after ischemia–reperfusion. Understanding these pathways may facilitate the development of therapies that replicate endotoxin’s cardioprotective effects while avoiding systemic inflammatory responses.

A key strength of this study is its comprehensive approach, integrating in vivo, ex vivo, and in vitro models to assess the effects of endotoxin on ischemia–reperfusion (I/R) injury. The in vivo experiments followed recent guidelines for experimental myocardial ischemia and reperfusion [45], utilizing a minimally invasive LAD ligation technique [46]. This method minimized surgical complications and contributed to higher survival rates. In the ex vivo experiments, isolated rat hearts were perfused with autologous blood, helping to maintain physiological perfusion parameters within optimal ranges. Moreover, normothermic perfusion was employed, a method known to enhance post-ischemic myocardial recovery compared with hypothermic perfusion techniques [47]. The in vitro component employed primary cardiomyocytes freshly isolated from neonatal rats. This approach offers clear advantages over immortalized or genetically modified cell lines, as primary cells more closely recapitulate in vivo physiological conditions, reducing experimental artifacts and lowering research costs. In the hypoxia/reoxygenation model, untreated groups exhibited reduced cardiomyocyte density, indicative of increased necrosis, which correlated with the greater extracellular matrix deposition observed in ischemia-reperfused hearts that had not received preconditioning. The recovery of cardiomyocyte density in cells pretreated with a nonlethal dose of endotoxin indicates enhanced myocardial protection or regenerative capacity following I/R injury. Our results demonstrated that cardiomyocytes treated with an endotoxin dose of 1 µg/mL exhibited the highest survival rate, whereas cells treated with lower doses of 0.5 and 0.1 µg/mL showed comparatively reduced survival. Notably, pretreatment with a higher endotoxin dose of 2 µg/mL led to a significant decline in cell viability. These findings indicate that lower doses of endotoxin provide minimal or no cardioprotective benefit, whereas higher doses can be detrimental, consistent with previous research demonstrating dose-dependent effects of endotoxin on cardiac cells [48]. Endotoxin-preconditioned cardiomyocytes exhibited significant preservation of mitochondrial respiration parameters, including basal and maximal respiration, spare respiratory capacity, and ATP production, while maintaining glycolytic activity. These findings indicate that endotoxin preconditioning stabilizes mitochondrial oxidative phosphorylation during reperfusion, thereby sustaining cellular energy balance. The reduced expression of hypoxia-inducible factor-1α (HIF-1α) further suggests alleviation of cellular hypoxic stress. In parallel, flow cytometry revealed a marked decrease in apoptotic cell death, demonstrating that endotoxin preconditioning provides robust anti-apoptotic protection. Collectively, these results support the hypothesis that mitochondrial stabilization is a central mechanism through which endotoxin preserves cardiomyocyte survival during reperfusion.

In contrast, our findings differ from those of Qiu et al., who reported that LPS endotoxin pre-exposure worsened hypoxia–reoxygenation injury in cardiomyocytes via a reactive oxygen species (ROS)-dependent mechanism [49]. Notably, their experiments were conducted under high-glucose conditions, which likely served as an aggravating factor, amplifying cellular damage during the inflammatory response induced by endotoxin preconditioning. These observations underscore the importance of considering metabolic factors, such as hyperglycemia, when assessing the effects of endotoxin pre-exposure on cellular function in ischemia–reperfusion models. A notable limitation of the present study is the potential confounding influence of anesthetic agents used during in vivo experiments. Gas anesthesia has been previously reported to exert cardioprotective preconditioning effects [50,51], which may have synergistically enhanced the endotoxin-induced antioxidative responses observed here. This overlap could have amplified the cardioprotective outcomes, limiting the ability to attribute the effects solely to endotoxin pretreatment. Additionally, the observed result may also reflect contributions from hypoxia-induced preconditioning. Hypoxic exposure is known to activate adaptive cellular mechanisms that reduce mitochondrial oxidative metabolism, thereby improving cell survival under low-oxygen conditions [51,52,53]. Such endogenous preconditioning may have partially contributed to the attenuation of oxidative stress and myocardial injury, representing an additional factor influencing the protective effects observed in endotoxin-preconditioned cardiomyocytes. Although pilot data supported the chosen sample size, the limited group size may reduce the statistical power to detect small differences. Therefore, these findings should be interpreted as exploratory, warranting further validation in larger cohorts.

### Clinical and Translational Implications

Although our findings underscore the potent cardioprotective effects of endotoxin preconditioning, translating these results into clinical practice poses a significant challenge. Endotoxin is a powerful immunological stimulus that, in septic conditions, can induce myocardial depression, systemic inflammation, and multiorgan dysfunction [52,53,54,55]. This dual nature renders endotoxin-based interventions inherently delicate, as harnessing its protective signaling while avoiding systemic toxicity is complex. The cardioprotective effects observed in controlled experimental settings may not fully translate to clinical contexts such as sepsis or endotoxemia. Future approaches may focus on targeting downstream mediators of endotoxin signaling, such as TLR4-dependent protective pathways, or controlled activation of endogenous preconditioning mechanisms to achieve myocardial protection without triggering systemic inflammatory responses. Despite the challenges and limitations inherent in the current study, our findings provide novel insights into the role of mitochondrial oxidative injury in the pathophysiology of septic cardiomyopathy, a condition that continues to require focused research efforts, and future studies employing large animal models to investigate endotoxin-induced cardioprotective effects during ischemia–reperfusion are essential. Such models better replicate human myocardial infarction physiology and will be crucial for translating experimental findings into clinically applicable therapies.

## 4. Materials and Methods

### 4.1. Animal Preparation

This study utilized fifteen adult male Sprague–Dawley (SD) rats (*n* = 5 per group), each weighing between 450 and 530 g. In addition, approximately 180 neonatal male and female rats (10 rats per experiment) were used for in vitro experiments. All animals were obtained from a certified vendor (Samtako Bio Korea Co., Ltd., Osan, Republic of Korea). Adult rats were housed in temperature-controlled cages, cleaned and disinfected daily, and maintained under a 12 h light–dark cycle with ad libitum access to standard rodent chow and water. Neonatal rats were kept with their mothers until the time of experimentation to ensure stable body temperature and adequate breastfeeding. All animal handling procedures and experimental protocols were conducted in compliance with the Guidelines for the care and use of laboratory animals at Chonnam National University Hospital.

### 4.2. Experimental Design and Endotoxin Pretreatment

**Figure 9:**
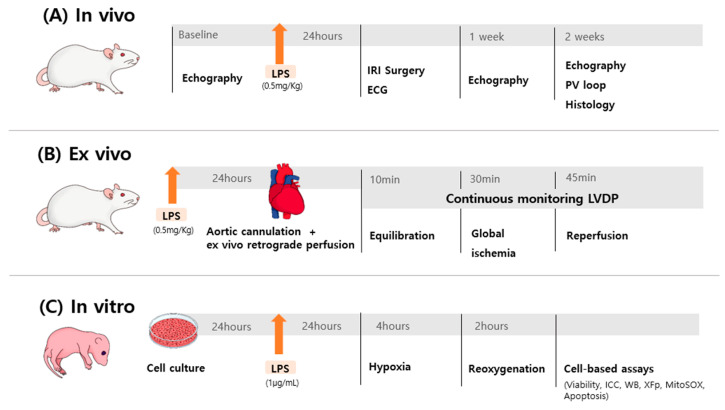
Experimental design and timeline for in vivo, ex vivo, and in vitro studies. (**A**) In vivo: Ischemia–reperfusion injury (IRI) was induced by temporary ligation of the left anterior descending (LAD) coronary artery for 30 min, followed by ligature removal to initiate reperfusion. ECG recordings were obtained before, during, and after LAD ligation (Supplementary Material, Figure S1). (**B**) Ex vivo: Global ischemia was induced by closing the aortic perfusion cannula for 30 min, followed by reopening to restore reperfusion. Left ventricular developed pressure (LVDP) was continuously monitored before, during, and after ischemia. (**C**) In vitro: Hypoxia was induced for 4 h, followed by 2 h of reoxygenation before performing cell-based assays. Abbreviations: LPS, lipopolysaccharide; PV loop, pressure–volume loop; LVDP, left ventricular developed pressure (mmHg); IC, immunocytochemistry; WB, Western blot; XFp, Seahorse XFp Mito Stress Test; MitoSOX, MitoSOX™ Green mitochondrial superoxide indicator.

For the in vivo and ex vivo studies, rats were randomly assigned to one of three groups (*n* = 5 per group). In the endotoxin-treated group, a single intraperitoneal dose of lipopolysaccharide (LPS; 0.5 mg/kg; Escherichia coli O127:B8, Difco Laboratories, Detroit, MI, USA) was administered 24 h prior to the ischemia–reperfusion procedure. The non-treated group received an equivalent volume of 0.9% normal saline 24 h before the experiment, and the sham group served as the control. For the in vitro studies, cultured cardiomyocytes were randomly divided into three groups. The control group consisted of untreated cells maintained under normoxic conditions. The hypoxia–reoxygenation (H/R) group consisted of untreated cells exposed to hypoxia followed by reoxygenation. The LPS-treated H/R group consisted of cells pretreated with LPS at different concentrations (0.1, 0.5, 1.0, and 2.0 μg/mL) before hypoxia and subsequent reoxygenation. LPS concentrations were selected based on previous studies [16,27], with minor modifications, including the addition of a higher dose (2.0 μg/mL) in the in vitro protocol to explore the optimal endotoxin concentration for myocardial protection under hypoxic–reoxygenation conditions. Group allocation was performed by an independent researcher who was not involved in data collection (Figure 9A–C). Investigators performing surgical procedures, echocardiography, ex vivo perfusion, histological analyses, mitochondrial respiration and other cell assays were blinded to group identity. Data analysis was conducted using coded datasets until statistical testing was completed.

### 4.3. In Vivo Studies

#### 4.3.1. Ischemia–Reperfusion Surgery

On the day of the experiment, rats were anesthetized via intramuscular injection of ketamine (80 mg/kg) and xylazine (8 mg/kg), followed by maintenance with inhaled isoflurane (1.5–2%, Baxter Health, Yellville, AR, USA) in 90% oxygen during surgery. After induction, animals were weighed, positioned supine on the operating table, and their limbs secured. Anesthetic depth was monitored and adjusted according to physiological parameters, including respiratory rate, heart rate, and body temperature. Orotracheal intubation was performed using a 16-gauge catheter, and mechanical ventilation was provided at 50 breaths/min with a tidal volume of 1.5 mL/100 g body weight (Harvard Volume Controlled Ventilator Model 683; Harvard Apparatus, Holliston, MA, USA). Rectal temperature was maintained at 37–38 °C using a heating pad. The left hemithorax was shaved, disinfected with alcohol, and prepped with povidone–iodine solution. A left thoracotomy was performed at the fourth intercostal space, and the ribs were gently retracted to expose the heart. The pericardium was opened, and the LAD was identified approximately 4 mm below the anterior–inferior edge of the left atrial appendage. LAD occlusion was achieved using a snare formed from 6-0 polypropylene suture and two micropipette tips (Supplementary Material, Video S1). Successful occlusion was confirmed by visual pallor of the left ventricular apex and characteristic electrocardiogram (ECG) changes (Supplementary Material, Figure S1B). After 30 min of ischemia, the snare was released to initiate reperfusion. The thoracic incision was closed in layers using 5-0 absorbable sutures. Postoperative care included administration of antibiotics and analgesics for three days. Animals were returned to their cages with free access to food and water during recovery.

#### 4.3.2. Echocardiography

Baseline transthoracic echocardiography was performed on all rats before the initiation of experimental protocols. Follow-up echocardiographic assessments were conducted at one and two weeks post–myocardial infarction (MI) induction using a VIVID 7 Dimension system (General Electric-Vingmed Ultrasound, Horton, Norway) equipped with a 12 MHz electronic transducer. Rats were anesthetized using the same protocol as for the I/R experiments (ketamine and xylazine for induction, followed by 2% isoflurane for maintenance). Following shaving and cleaning of the chest, animals were positioned supine, and the transducer was applied directly to the shaved chest wall. Images were acquired from the left parasternal short-axis view of the left ventricle (LV) (Figure 1A). Left ventricular systolic function was assessed by measuring left ventricular ejection fraction (LVEF) and fractional shortening (FS) in M-mode echocardiography. LVEF was calculated as the percentage change in left ventricular volume between end-diastole and end-systole, providing an index of global systolic performance. Fractional shortening, representing the percentage change in LV diameter between diastole and systole, was used as an additional measure of contractile function. In addition to systolic parameters, diastolic function was evaluated by measuring left ventricular end-diastolic dimension (LVEDD) and left ventricular end-systolic dimension (LVESD). These dimensions were obtained by tracing the internal diameter of the LV at the level of the papillary muscles. All measurements were recorded three times and averaged before statistical analysis. Echocardiographic procedures and calculations were performed following previously published protocols [56].

#### 4.3.3. In Vivo Hemodynamics

Two weeks following LAD ligation, hemodynamic parameters and indices of systolic and diastolic function were assessed using pressure-volume (PV) loop analysis in control (sham), ischemia/reperfusion (I/R), and endotoxin-treated groups. Measurements were acquired with Millar PV Systems MPVS Ultra (Millar Instruments, Inc., Pearland, TX, USA). Rats were anesthetized and maintained on 1–2% isoflurane throughout the procedure. A left thoracotomy was performed to expose the heart, and a 24-gauge needle was used to create a small stab wound in the ventricular apex. Through this access, a pressure-volume catheter was advanced into the left ventricle. The catheter position was adjusted to obtain stable, regular, rectangular-shaped PV loops, and signals were allowed to stabilize for approximately 10 min before data acquisition. Simultaneous pressure and volume measurements were recorded continuously for 1 h in all groups. Body temperature, fluid balance, and anesthesia depth were carefully monitored and maintained throughout the experiment. The following cardiac parameters were extracted from the PV loops: end-diastolic volume (EDV), end-systolic volume (ESV), end-diastolic pressure (EDP), end-systolic pressure (ESP), stroke work (SW), potential energy (PE), and pressure-volume area (PVA). After data collection, calibration of raw conductance volume signals was performed using cuvette calibration with fresh, heparinized warm blood and hypertonic saline calibration to derive absolute ventricular volumes. Hearts were subsequently excised and prepared for histological analysis. Pressure-volume area (PVA) was calculated using recorded SW and PE values in Microsoft Excel. PVA values were plotted against corresponding EDV measurements, and linear trendlines were fitted to illustrate the relationship between myocardial energetic demand and ventricular filling volume.

#### 4.3.4. Histology

Following hemodynamic measurements, hearts were excised and perfused with 40 mL of cold cardioplegic solution to arrest cardiac activity. Cross-sectional tissue samples were obtained from the border zone of the infarcted region. Samples were fixed in 3.7% formalin for 24 h at room temperature, then processed for paraffin embedding. Sections 4 μm thick were prepared and mounted on slides for Hematoxylin and Eosin (H&E) and Masson’s Trichrome staining. Stained slides were scanned using a Zeiss Axio Scan.Z1 slide scanner (Zeiss, Jena, Germany). For each heart, three non-overlapping sections from equivalent anatomical levels were analyzed. Within each section, fibrosis quantification was performed in five randomly selected high-power fields (HPFs) encompassing both infarcted and peri-infarct areas. Myocardial fibrosis was expressed as the percentage of blue-stained collagen area relative to the total myocardial area, calculated using ImageJ software (version 1.54d; National Institutes of Health, Bethesda, MD, USA) with color deconvolution analysis.

### 4.4. Ex Vivo Study

#### Isolated Normothermic Perfused Rat Heart Model

A total of 15 rats were allocated to the isolated perfused heart experiments. On the day before surgery, rats in the treatment group (*n* = 5) received a single intraperitoneal dose of endotoxin, identical to that used in the in vivo experiments. Rats in the ischemia–reperfusion (I/R) group (*n* = 5) received an equivalent volume of normal saline, while no injections were administered to the control (sham) group (*n* = 5). Twenty-four hours after treatment, animals were anesthetized, intubated, and the abdominal cavity was opened to expose the abdominal aorta, which was cannulated with a 20-G catheter. Approximately 10 mL of blood was withdrawn from each animal, followed by the infusion of approximately 40 mL of cold cardioplegic solution at 80 mL/min using an automatic syringe pump. The collected blood was introduced into the ex vivo perfusion circuit and maintained at constant volume flow. Following induction of cardiac arrest, the chest was opened, and the hearts were excised and mounted on a Langendorff perfusion apparatus, allowing retrograde aortic perfusion under constant pressure conditions. A compliant balloon was inserted into the left ventricle for continuous measurement of left ventricular developed pressure (LVDP). After a 10-min stabilization period, the aortic inflow was clamped to induce 30 min of global ischemia, followed by 45 min of reperfusion under normothermic conditions. LVDP was recorded throughout the procedure, along with heart rate (HR) and maximal and minimal first derivatives of ventricular pressure (±dP/dt). At the conclusion of perfusion, heart weight was measured, and coronary flow (CF) was calculated. This isolated heart preparation provided a highly controlled environment for evaluating intrinsic myocardial performance in the absence of systemic neurohumoral influences, allowing for precise assessment of contractile function, coronary hemodynamics, and ischemia–reperfusion injury. The incorporation of autologous blood into the circuit preserved physiological oxygen-carrying capacity and buffering properties, while normothermic perfusion closely approximated in vivo metabolic conditions. In addition, the standardized global ischemia protocol enabled reproducible evaluation of post-ischemic myocardial recovery and facilitated investigation of the potential cardioprotective effects of endotoxin treatment.

### 4.5. In Vitro Studies

#### 4.5.1. Primary Culture of Neonatal Rat Cardiomyocytes

Primary cardiomyocyte cultures were prepared from 1 to 3-day-old male and female Sprague–Dawley (SD) rats. After surface sterilization with 70% ethanol, pups were decapitated, and hearts were rapidly excised. Hearts were immediately rinsed in ice-cold buffer containing 116 mM NaCl, 20 mM HEPES, 10 mM NaH_2_PO_4_, 5.5 mM glucose, 5 mM KCl, and 0.8 mM MgSO_4_, adjusted to pH with NaOH. Myocardial tissue was minced into small fragments (1–3 mm^3^) and subjected to 5–6 cycles of enzymatic digestion in 0.4 mg/mL collagenase type II (Gibco, Waltham, MA, USA) and 0.6 mg/mL pancreatin (Gibco, USA) for 10–15 min at 37 °C with gentle shaking. After each digestion, supernatants were mixed (1:1) with ice-cold fetal bovine serum (FBS; 20%, Gibco, USA) and centrifuged at 1000 rpm for 5 min. Cell pellets were resuspended in DMEM supplemented with FBS, horse serum (Gibco, USA), 100 U/mL penicillin, and 100 μg/mL streptomycin (Gibco, USA). To enrich for cardiomyocytes, pre-plating was performed by incubating cells for 1 h at 37 °C in a humidified atmosphere (5% CO_2_, 95% air) to allow preferential attachment of non-cardiomyocytes. Following purification, viable cells were counted using a hemocytometer (Thermo Fisher Scientific, Waltham, MA, USA) and plated at the desired density. To inhibit fibroblast proliferation, 0.1 mM 5-bromo-2′-deoxyuridine (BrdU; Sigma–Aldrich, St. Louis, MO, USA) was added to the plating medium. Twenty-four hours after seeding, cardiomyocytes were allocated into three experimental groups before hypoxia–reoxygenation assays.

#### 4.5.2. Identification of Cultured Cardiomyocytes

Twenty-four hours after seeding, cells were assessed to confirm cardiomyocyte identity. Under bright-field microscopy (Philips, Inc., Cambridge, MA, USA), most cells exhibited firm adhesion to the culture substrate and demonstrated rhythmic, spontaneous contractions indicative of functional cardiac muscle cells (Supplementary Material, Video S2). Immunocytochemical staining for sarcomeric α-actinin, a definitive cardiomyocyte marker, revealed that more than 95% of cells were positively labeled (Figure 4), confirming high culture purity. Quantitative morphometric analysis using ImageJ software version 1.54d (National Institutes of Health, USA) further characterized the cells, measuring parameters such as cell size, density, sarcomere length, aspect ratio, and striation regularity, which collectively supported their identification as structurally organized cardiomyocytes.

#### 4.5.3. Hypoxia–Reoxygenation Protocol for Cultured Cardiomyocytes

Twenty-four hours after pretreatment with LPS, cultured neonatal rat cardiomyocytes were subjected to a standardized hypoxia–reoxygenation (H/R) protocol. Hypoxia was induced using a modular incubator chamber (MIC-101; Billups-Rothenberg, Inc., San Diego, CA, USA) connected to a certified gas mixture containing 1% O_2_, 5% CO_2_, and balance N_2_ (Dae Deok Gas Co., Ltd., Incheon, Republic of Korea). The chamber atmosphere was equilibrated by continuous flushing with the hypoxic gas at 20 L/min for 7–10 min, with inlet pressure maintained at 20 psi. This purging procedure reliably reduced the chamber pO_2_ to <15 mmHg, as specified by manufacturer calibration curves. The sealed chamber was then placed in a humidified CO_2_ incubator (37 °C; 5% CO_2_). To sustain hypoxic conditions, an additional purge was performed at the 2-h mark. Total hypoxic exposure was maintained for 4 h. Reoxygenation was achieved by transferring the cultures to normoxic incubator conditions (95% air, 5% CO_2_) for 2 h, ensuring rapid restoration of atmospheric pO_2_ levels.

#### 4.5.4. Cell Viability Analysis

Following reoxygenation, cell viability was assessed using the MTT assay. Cardiomyocytes cultured in 96-well plates were incubated for 4 h at 37 °C in the dark with 20 µL of 3-(4,5-dimethylthiazol-2-yl)-2,5-diphenyltetrazolium bromide (MTT) solution (5 mg/mL; Thermo Fisher Scientific, USA). Subsequently, 50 µL of dimethyl sulfoxide (DMSO; Thermo Fisher Scientific, USA) was added to each well to dissolve the formazan crystals. Plates were gently shaken for 10 min at room temperature, and absorbance was measured at 490 nm using a spectrophotometer (Thermo Fisher Scientific, USA).

#### 4.5.5. Immunocytochemical Evaluation of Hypoxia

Cells were fixed with 4% paraformaldehyde (PFA) for 15 min and washed three times with phosphate-buffered saline (PBS). Permeabilization was performed using 0.1% Triton X-100 for 15 min, followed by overnight incubation at 4 °C with primary antibody against hypoxia-inducible factor 1-alpha (HIF-1α; Invitrogen, Waltham, MA, USA, ESEE122) diluted 1:200 in blocking solution (1% BSA). Cells were then incubated with Alexa Fluor 594-conjugated secondary antibody (1:500) for 1 h at room temperature in the dark. After three PBS washes, slides were mounted with medium containing DAPI (Invitrogen, USA) and visualized under confocal microscopy (Figure 6A). A similar procedure was performed during the cell identification and characterization step (Figure 4), replacing the primary antibody with sarcomeric α-actinin (Sigma, A7811).

#### 4.5.6. Western Blot Analysis of Hypoxia-Related Protein Expression

Cells (6 × 10^5^ cells/well) were lysed on ice using radioimmunoprecipitation assay (RIPA) buffer (Thermo Fisher Scientific, USA) supplemented with a protease inhibitor cocktail (Thermo Fisher Scientific, USA). Protein concentrations were determined using the Pierce bicinchoninic acid (BCA) Protein Assay Kit (Thermo Fisher Scientific, USA). Equal amounts of protein (30 µg) were separated by 8% sodium dodecyl sulfate–polyacrylamide gel electrophoresis (SDS-PAGE) and transferred onto polyvinylidene fluoride (PVDF) membranes (Bio-Rad, Hercules, CA, USA). Membranes were blocked and incubated overnight at 4 °C with primary antibodies against hypoxia-inducible factor 1-alpha (HIF-1α; 1:500; Sigma-Aldrich) and beta-actin (1:5000; Abcam, Cambridge, UK). The following day, membranes were incubated with horseradish peroxidase (HRP)-conjugated secondary antibody (1:2000; Sigma-Aldrich) for 1 h at room temperature. Protein bands were visualized using a chemiluminescence detection system (Supplementary Material, Western Blot). Quantitative analysis of band intensities was performed using ImageJ software version 1.54d (National Institutes of Health, USA), and statistical analyses were conducted with GraphPad Prism version 9.0.

#### 4.5.7. Evaluation of Mitochondrial Respiration

Mitochondrial function in cultured cardiomyocytes was assessed in real time by measuring extracellular flux using the Seahorse Bioscience XFp Extracellular Flux Analyzer (Seahorse Bioscience, North Billerica, MA, USA), following previously established protocols [57,58]. The XFp Cell Mito Stress Test Kit was employed to determine the oxygen consumption rate (OCR), an indicator of mitochondrial respiration. Additionally, the XFp Glycolysis Stress Test Kit was used to measure the extracellular acidification rate (ECAR), reflecting the glycolytic capacity of the cells. OCR measurements were performed under basal conditions, followed by sequential injections of mitochondrial modulators: oligomycin (0.5 μM), carbonyl cyanide p-trifluoromethoxyphenylhydrazone (FCCP, 1 μM), and a combination of rotenone (1 μM) and antimycin A (1 μM). These agents allowed assessment of basal respiration, maximal respiration, ATP production, and spare respiratory capacity, the latter indicating the cell’s ability to meet increased energetic demand. Experiments were conducted in triplicate with at least three independent biological replicates. Data were analyzed using Seahorse Wave software (version 2.6), and statistical comparisons were performed with GraphPad Prism version 9.0.

#### 4.5.8. Measurement of Mitochondrial Reactive Oxygen Species (ROS)

MitoSOX™ Green mitochondrial superoxide indicator (Thermo Fisher Scientific, M36005, Eugene, OR, USA) was used to selectively detect mitochondrial superoxide in live cardiomyocytes. A 5 mM stock solution of MitoSOX™ Green was prepared by dissolving the reagent vial contents in 13 µL of anhydrous dimethyl sulfoxide (DMSO). The working solution (500 nM) was made by diluting 5 µL of the 5 mM stock into 50 mL of Hank’s Balanced Salt Solution (HBSS). Four milliliters of the working solution were added to cells cultured in 60 mm dishes, which were then incubated at 37 °C with 5% CO_2_ for 30 min. Following incubation, cells were washed three times with warm HBSS. Live-cell fluorescence imaging was performed using a fluorescence microscope (excitation/emission: 510/580 nm) under identical exposure settings across samples. Fluorescence intensity was quantified using ImageJ software version 1.54d (National Institutes of Health, USA). Experiments were conducted in triplicate with at least three independent biological replicates.

#### 4.5.9. Analysis of Cellular Apoptosis

Apoptosis induced by hypoxia–reoxygenation injury was evaluated using the APC Annexin V Apoptosis Detection Kit in combination with Propidium Iodide (BioLegend, San Diego, CA, USA). Cells were washed twice with cold staining buffer (BioLegend, USA) and resuspended in Annexin V binding buffer at a density of 2 × 10^5^ cells/mL. Approximately 100 µL of the cell suspension was transferred into 5 mL test tubes, to which 2 µL of APC Annexin V and 3 µL of Propidium Iodide solution were added simultaneously. The mixture was gently vortexed and incubated at room temperature in the dark for 15 min. Subsequently, 400 µL of Annexin V binding buffer was added to each tube corresponding to the control, hypoxia–reoxygenation (H/R), and H/R + LPS groups. Apoptotic cells were detected using a flow cytometer (Beckman Coulter, Brea, CA, USA). Forward and side scatter plots were used to gate viable cells, and quadrants were set based on unstained and single-stained controls to distinguish live, early apoptotic, late apoptotic, and necrotic cells. Experiments were performed in triplicate with at least three independent biological replicates. Data were analyzed with Kaluza software version 2.2.1, and statistical comparisons were performed using GraphPad Prism.

#### 4.5.10. Statistical Analysis

All statistical analyses were performed using GraphPad Prism version 9.0 software (GraphPad Software, San Diego, CA, USA). Data were tested for normality using the Shapiro–Wilk test and for homogeneity of variances using Levene’s test. Comparisons involving multiple groups were conducted using one-way analysis of variance (ANOVA) followed by Tukey’s post hoc test to perform pairwise comparisons and identify specific group differences. All tests were two-tailed, and a *p*-value of less than 0.05 was considered statistically significant. Results are presented as mean ± standard deviation (SD) to convey variability within each experimental group. In vivo and ex vivo experiments were conducted in quintuplicate (*n* = 5), and in vitro experiments were performed in triplicate (*n* = 3). Sample sizes (*n* = 3–5 per group) were guided by pilot experiments conducted under the same experimental conditions, which showed reproducible trends and acceptable variability in the primary outcome (mitochondrial oxygen consumption). Given the invasive nature of the procedures, group sizes were minimized in accordance with the principles of the 3Rs (Replacement, Reduction, and Refinement) and institutional ethical guidelines. Graphical representations were prepared using Prism, with error bars reflecting standard deviation unless otherwise noted. Statistical significance is indicated as follows: ns (not significant), * (*p* < 0.05), ** (*p* < 0.01), and *** (*p* < 0.001), relative to the control group unless otherwise specified.

## 5. Conclusions

In summary, this study demonstrates that endotoxin preconditioning significantly mitigates myocardial injury induced by ischemia–reperfusion by preserving left ventricular function, reducing fibrosis and inflammation, and attenuating oxidative stress and apoptosis through maintenance of mitochondrial respiration. These findings highlight the potential translational value of endotoxin-derived preconditioning pathways for protecting the heart against reperfusion injury. Nonetheless, careful consideration of dose and systemic effects will be critical before clinical translation. While restoring blood flow to the ischemic myocardium remains the cornerstone of limiting myocardial damage, reperfusion itself can paradoxically exacerbate injury, as reflected in both our results and previous studies. This underscores the importance of exploring myocardial preconditioning strategies as promising interventions to safeguard the heart during reperfusion therapy.

## Data Availability

The raw data supporting the conclusions of this article will be made available by the authors on request.

## Supplementary Materials

The following supporting information can be downloaded at: https://www.mdpi.com/article/10.3390/ijms262211162/s1.
