# Supplementary material for: Endotoxin Pretreatment Mitigates Myocardial Ischemia-Reperfusion Injury Through Preservation of Mitochondrial Respiration: A Combined Assessment of In Vivo, Ex Vivo, and In Vitro Data
Source: Int J Mol Sci. 2025 Nov 19;26(22):11162. doi: 10.3390/ijms262211162 (PMC12653926; doi:10.3390/ijms262211162)
Supplement: Supplementary file 1 [file ijms-26-11162-s001.zip › ijms-3958702-supplementary.pptx]

## Slide 1
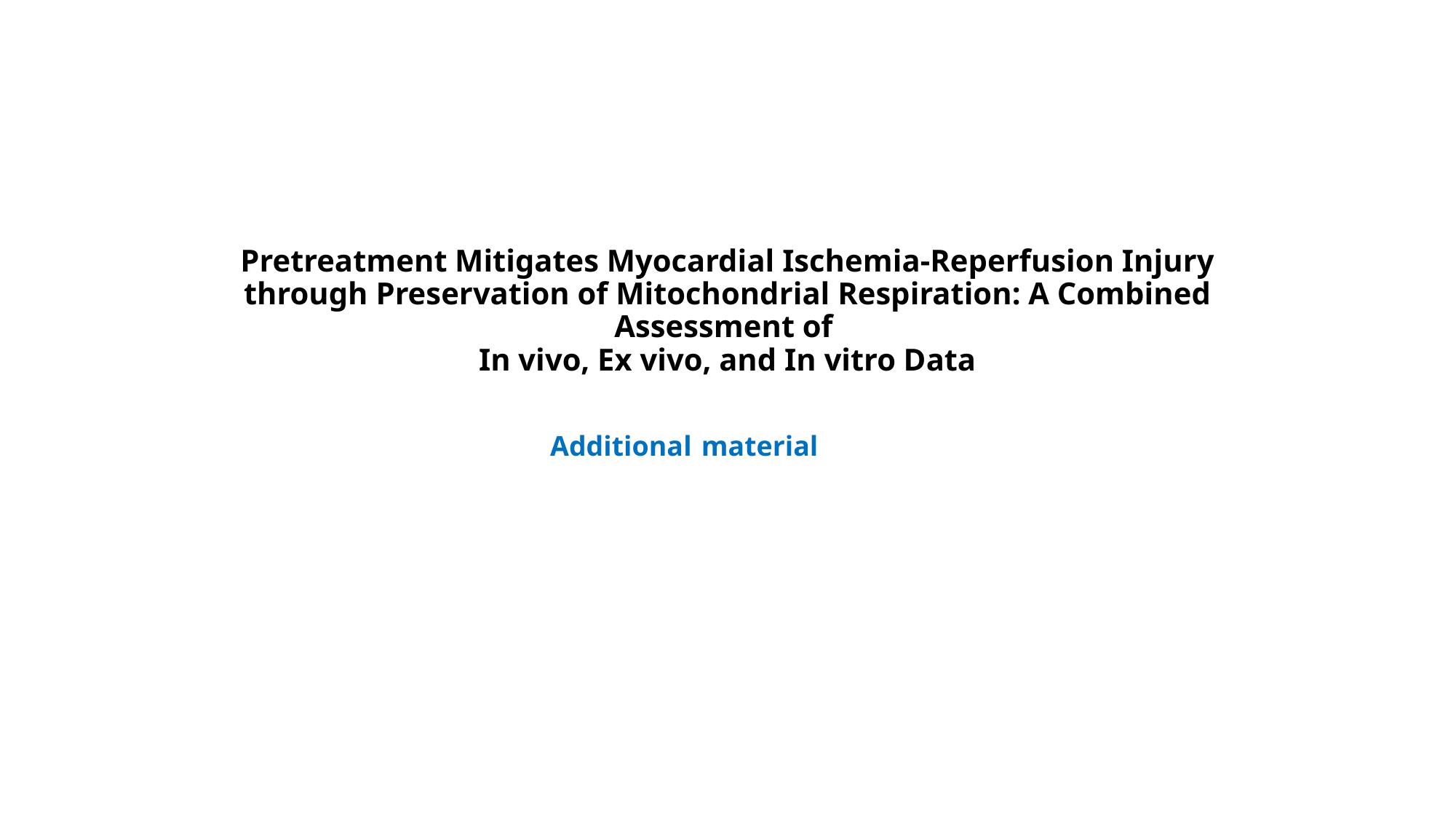

# Pretreatment Mitigates Myocardial Ischemia-Reperfusion Injury through Preservation of Mitochondrial Respiration: A Combined Assessment of In vivo, Ex vivo, and In vitro Data
Additional material

## Slide 2
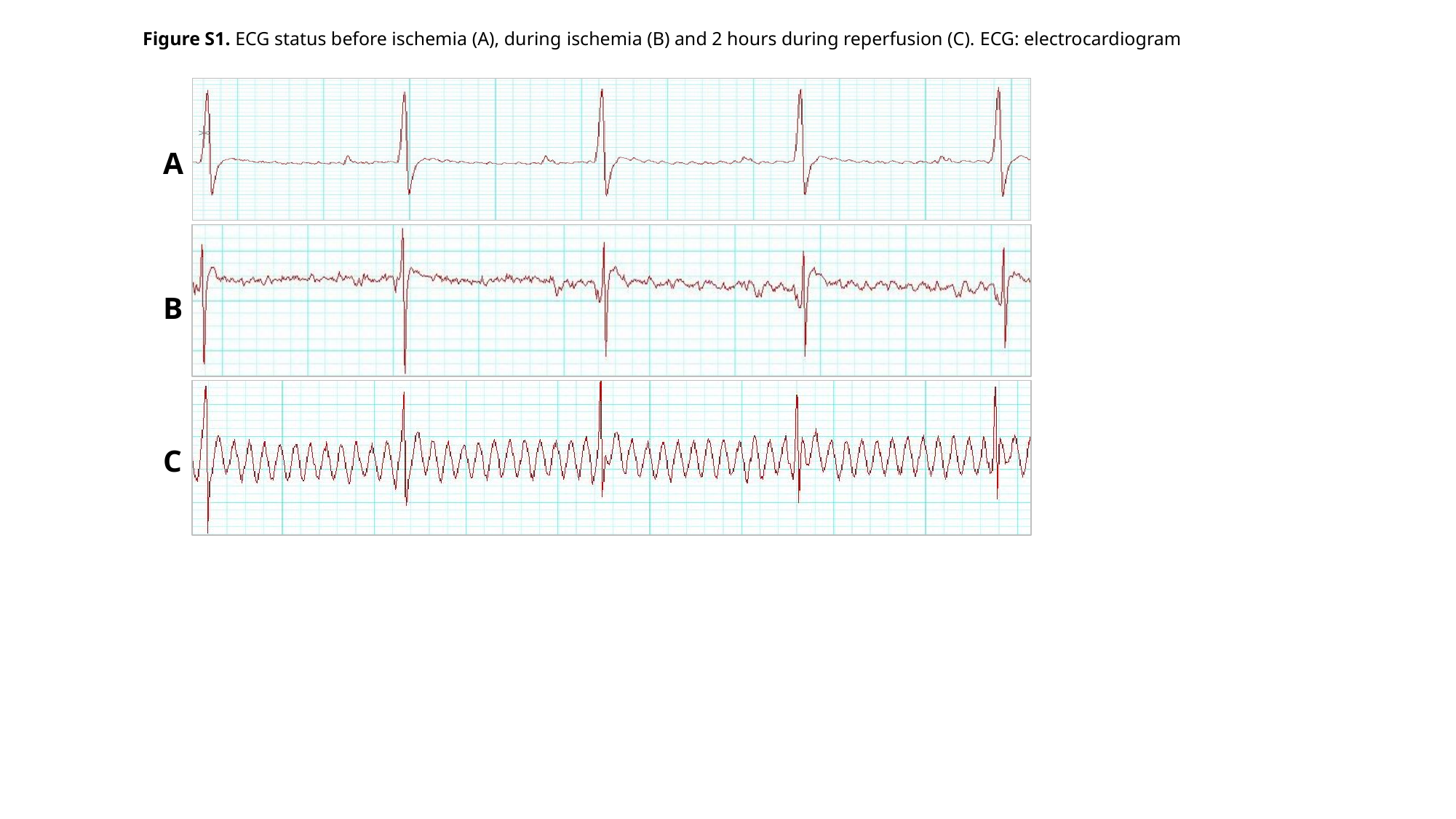

Figure S1. ECG status before ischemia (A), during ischemia (B) and 2 hours during reperfusion (C). ECG: electrocardiogram
A
B
C

## Slide 3
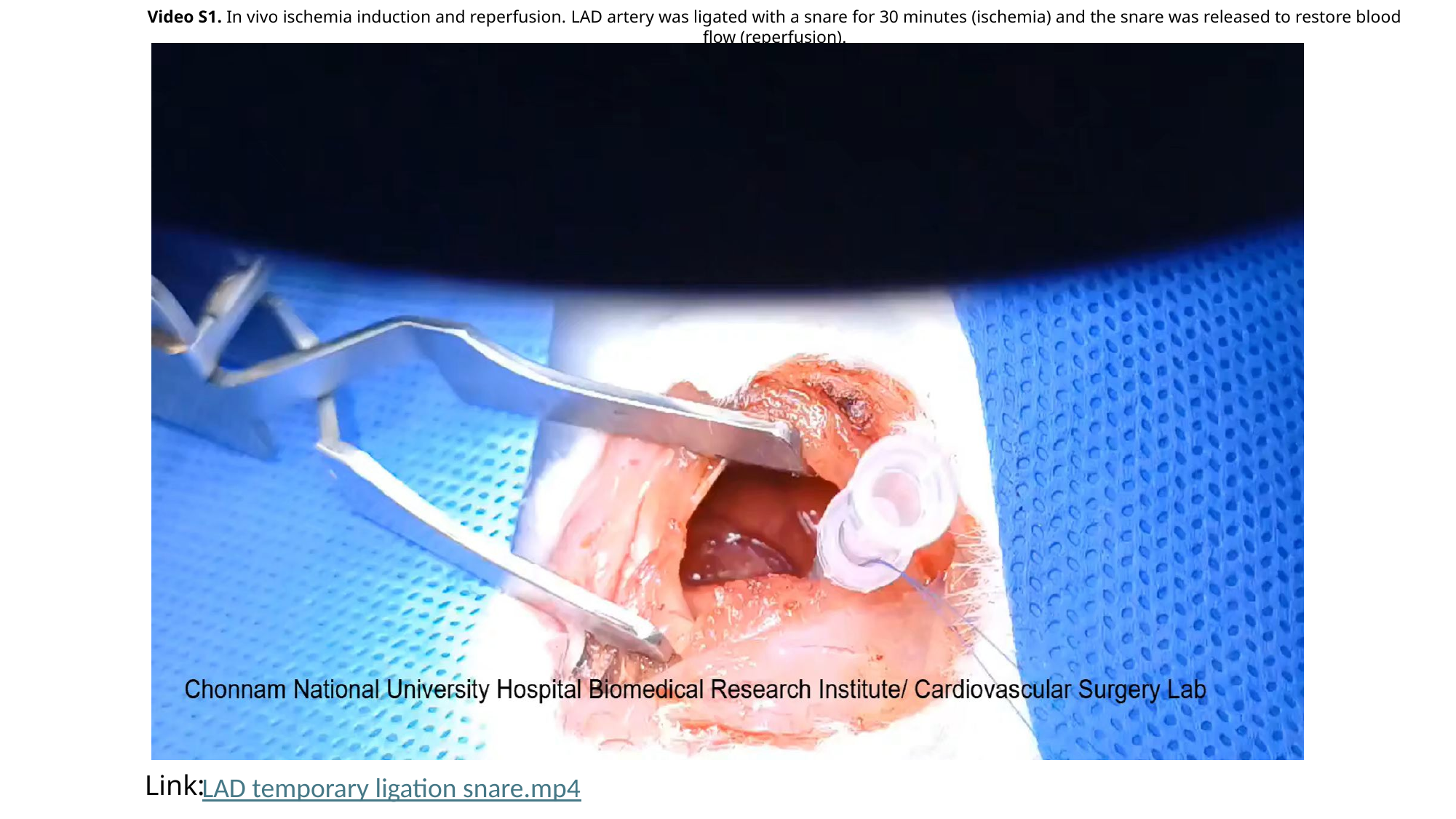

Video S1. In vivo ischemia induction and reperfusion. LAD artery was ligated with a snare for 30 minutes (ischemia) and the snare was released to restore blood flow (reperfusion).
Link:
LAD temporary ligation snare.mp4

## Slide 4
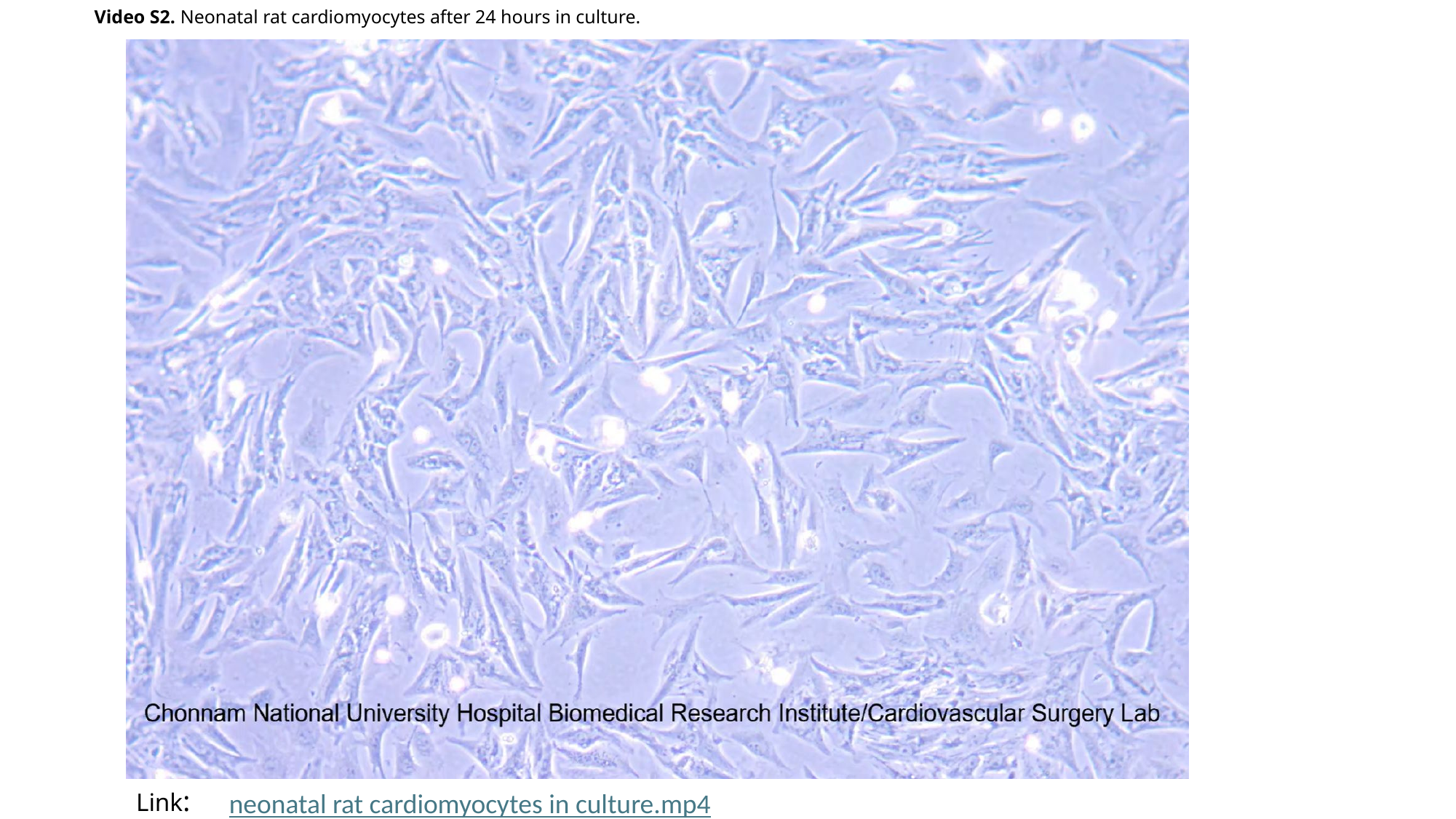

Video S2. Neonatal rat cardiomyocytes after 24 hours in culture.
Link:
neonatal rat cardiomyocytes in culture.mp4

## Slide 5
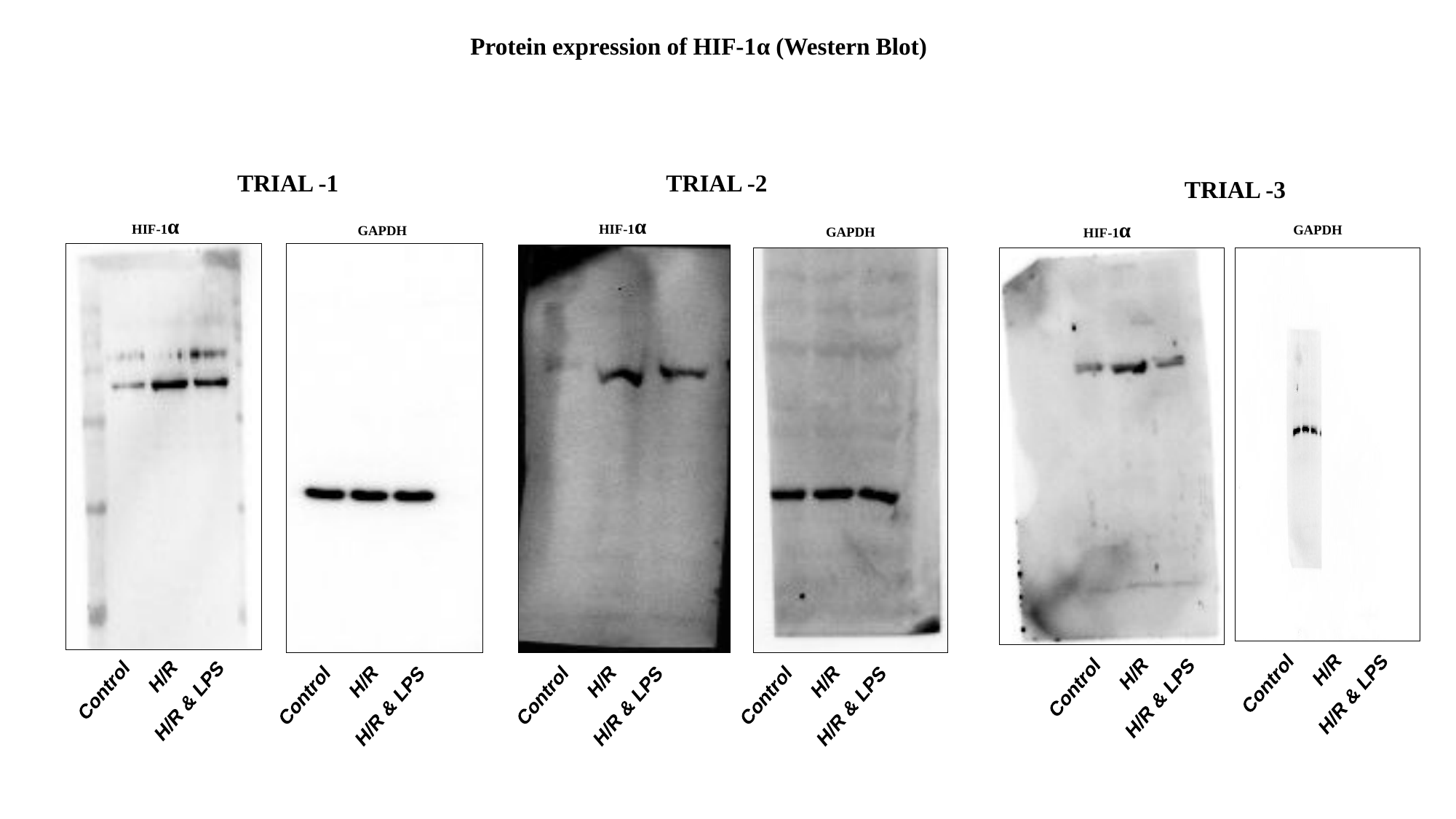

Protein expression of HIF-1α (Western Blot)
TRIAL -1
TRIAL -2
TRIAL -3
HIF-1α
HIF-1α
HIF-1α
GAPDH
GAPDH
GAPDH

## Slide 6
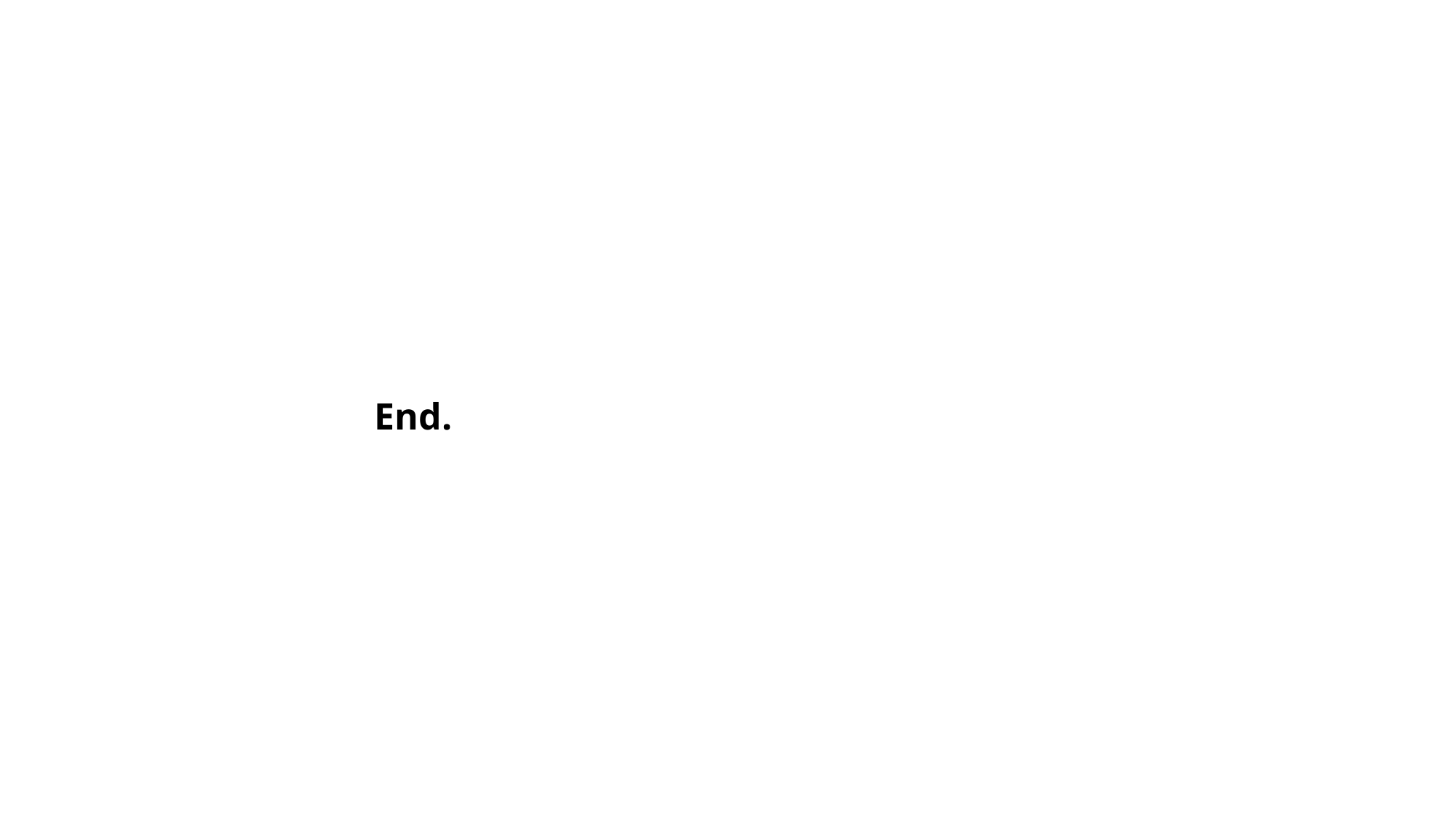

End.
